# Supplementary material for: Spatiotemporal regulation of the GPCR activity of BAI3 by C1qL4 and Stabilin-2 controls myoblast fusion
Source: Nat Commun. 2018 Oct 26;9:4470. doi: 10.1038/s41467-018-06897-5 (PMC6203814; doi:10.1038/s41467-018-06897-5)
Supplement: Supplementary file 1 — Supplementary information [file 41467_2018_6897_MOESM1_ESM.pdf]

Hamoud et al.

Spatiotemporal regulation of the GPCR activity of BAI3 by C1qL4 and Stabilin-2 controls myoblast fusion

Supplementary Information

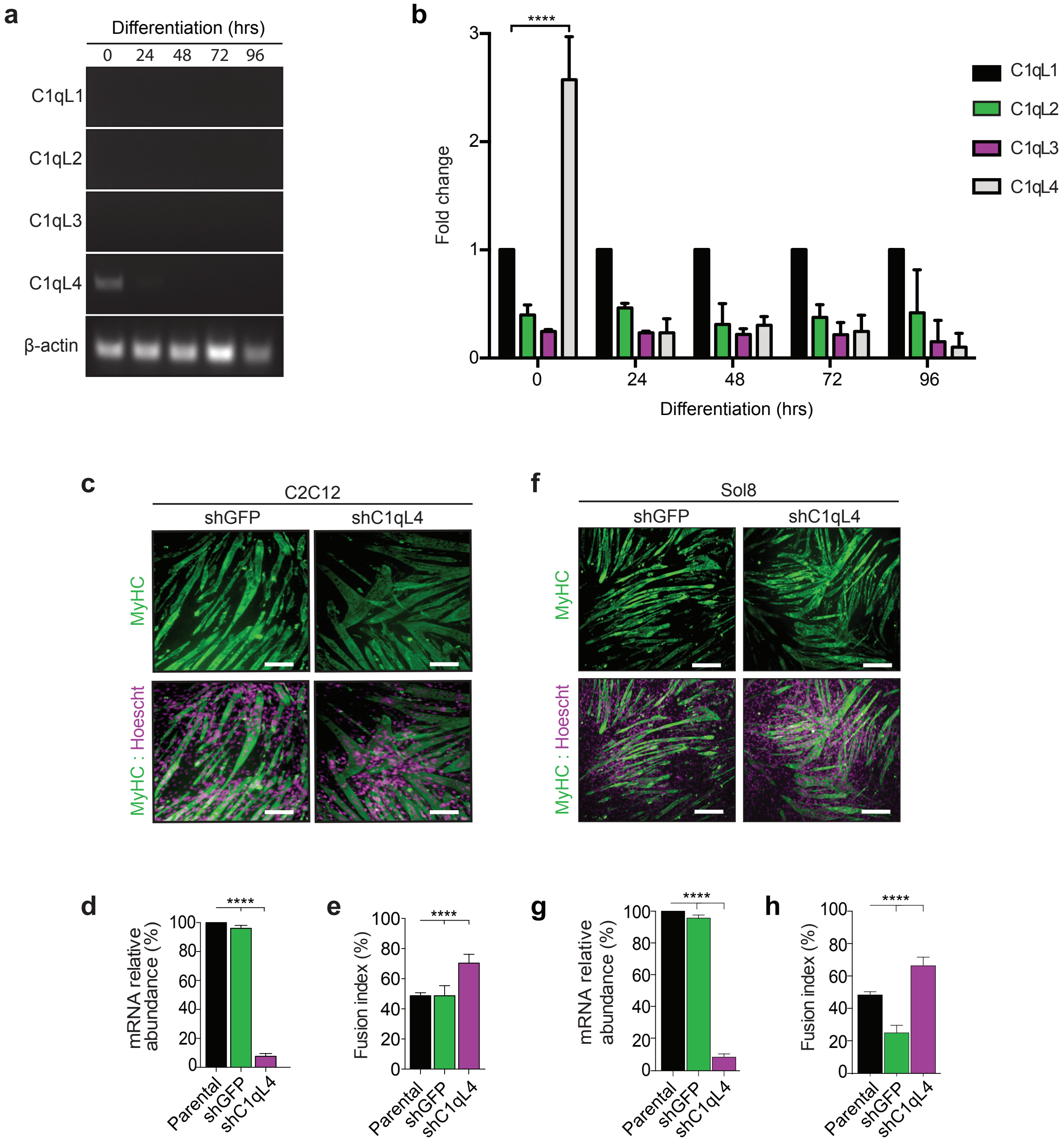

Supplementary Figure 1. C1qL4 family proteins inhibit myoblast fusion. (a) Expression levels of C1qL1-4 mRNAs during C2C12 myoblasts differentiation (0-96hrs) was measured by semi-quantitative RT-PCR. β-Actin was used as a loading control. **(b)** Real-Time Quantitative RT-PCR of C1qL1-4 mRNA expression throughout C2C12 differentiation. (**c-h)** C2C12 or Sol8 expressing an empty vector (GFP) or an shRNA targeting C1qL4 were generated by retroviral infections. **c, f)** Downregulation of C1qL4 increases myoblast fusion after 48 hours of differentiation in both cell types. (**d, g)** Real-time Q-RT-PCR amplifications were performed to confirm the specific knockdown of C1qL4. **(e, h)** Quantifications of experiments shown in **(c, f)**. Myofibers were stained for Myosin Heavy Chain (MyHC, MF20 antibody (green)) and nuclei were revealed by Hoechst (purple). Error bars indicate standard deviation. Scale bar=100um. One-way ANOVA followed by a Bonferroni test was used to calculate the p values; ****$P<0.0001$.

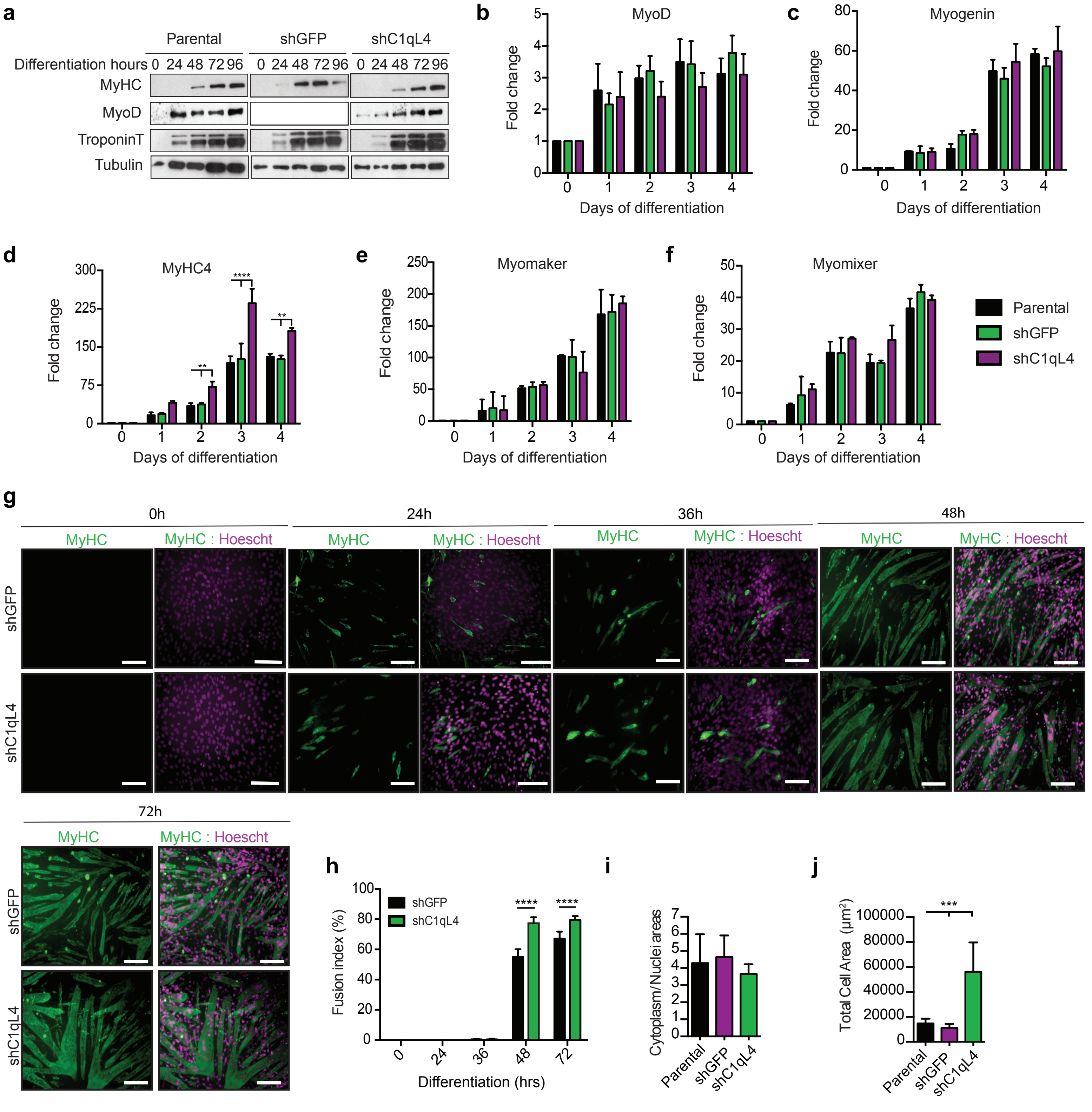

Supplementary Figure 2. C1qL4 knockdown has no effect on myoblast differentiation. (a) Depletion of C1qL4 does not affect C2C12 differentiation (from 24 to 96 hrs of differentiation). **(b-d)** Real-time Q-RT-PCR amplifications of differentiation markers were performed to study the effect of C1qL4 knockdown on MyoD **(b)**, Myogenin **(c)** Myosin Heavy Chain 4 (MyHC4) **(d)**. **(e-f)** Real-time Q-RT-PCR amplifications were performed to study the effect of C1qL4 knockdown on Myomaker **(e)** and Myomixer **(f)** mRNAs expression. **(g)** Similar rates of cell fusion were observed between control cells and shC1qL4 cells. Increased fusion was observed at 48 hrs of differentiation but not at t=0 or t=24 hrs. **(h)** Quantification of experiments shown in **(g)**. **(i-j)** C1qL4 knockdown does not lead to hypertrophy. Quantification of the fibers cytoplasm area/nuclei area ratio **(i)** and total cell area **(j).** Myofibers were stained for Myosin Heavy Chain (MyHC, MF20 antibody (green)) and nuclei were revealed by Hoechst (purple). Error bars indicate standard deviation. Scale bar=100um. One-way ANOVA followed by a Bonferroni test was used to calculate the p values; **P<0.01,***P<0.001,****P<0.0001.

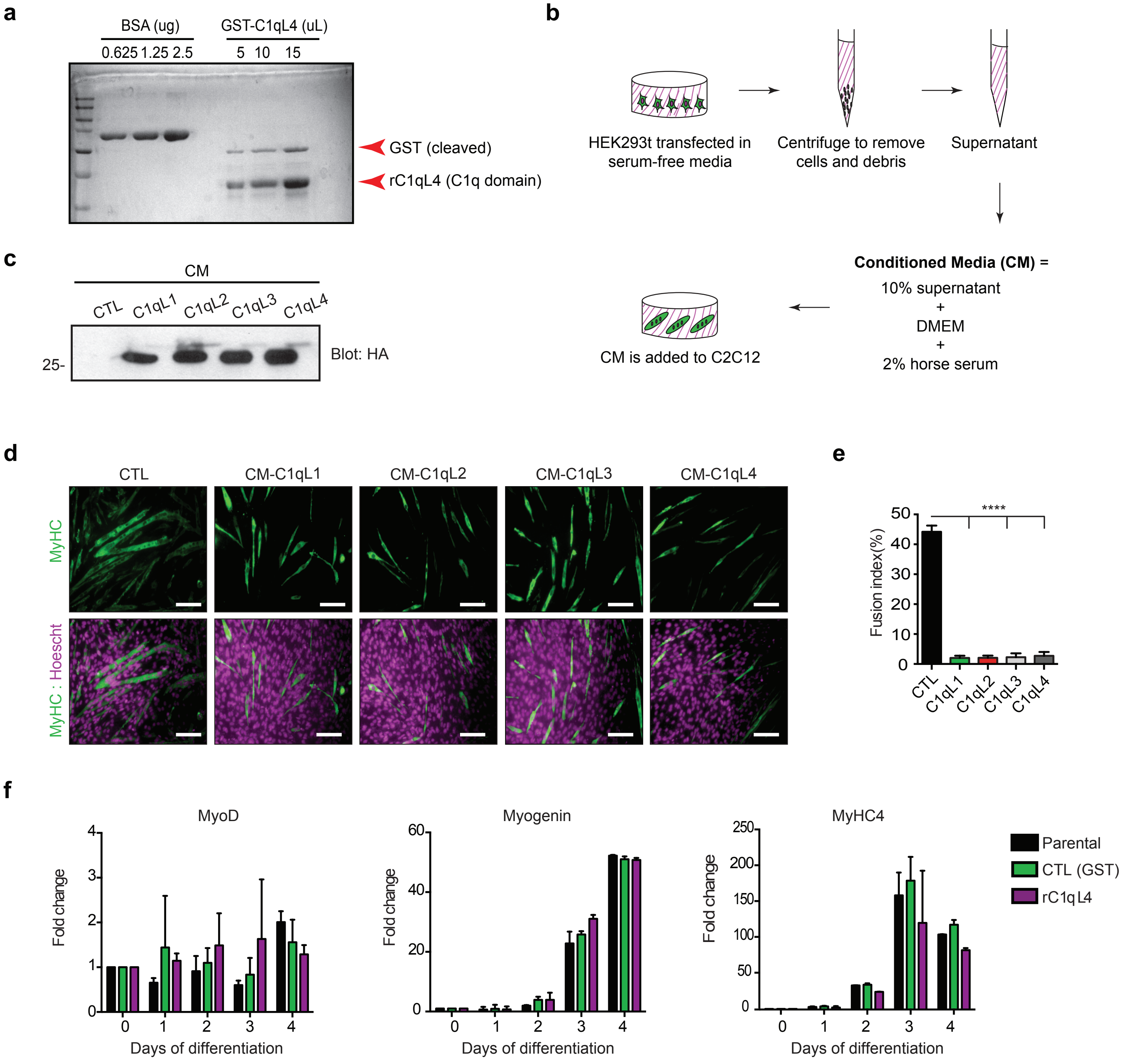

Supplementary Figure 3. C1qL1-4 inhibits myoblast fusion. (a) Example of recombinant GST-C1qL4 (C1q domain) purified from bacteria and the cleavage of the GST tag by Thrombin. SDS-PAGE gel analysis followed by Coomassie staining to assess protein integrity. Such gel was used to determine the concentration of rC1qL4 and approximately 100ng per ml of differentiation media was used in C2C12 cells differentiation assays. **(b-e)** C1qL-family members are able to inhibit myoblast fusion. **b)** Schematic representation of how the conditioned media containing various secreted proteins (HA-C1qLs and BAI3 fragments later in the paper) is made and how this media was used to prepared differentiation media containing the indicated proteins. **c)** C1qL-family members expression in conditioned media: C1qL1, C1qL2, C1qL3 and Cq1L4. **(d)** Parental C2C12 cells treated with conditioned media show that C1qL1-4 inhibits fusion. **(e)** Quantification of experiments shown in **(d)**. **(f)** Real-time Q-RT-PCR was performed to study the effect of rC1qL4 treatment on C2C12 expression of the following differentiation markers mRNAs: MyoD, Myogenin, MyHC4. No significant difference was observed during 4 days of differentiation. Myofibers were stained for Myosin Heavy Chain (MyHC, MF20 antibody (green)) and nuclei were revealed by Hoechst (purple). Error bars indicate standard deviation. Scale bar=100um. One-way ANOVA followed by a Bonferroni test was used to calculate the p values; ****P<0.0001.

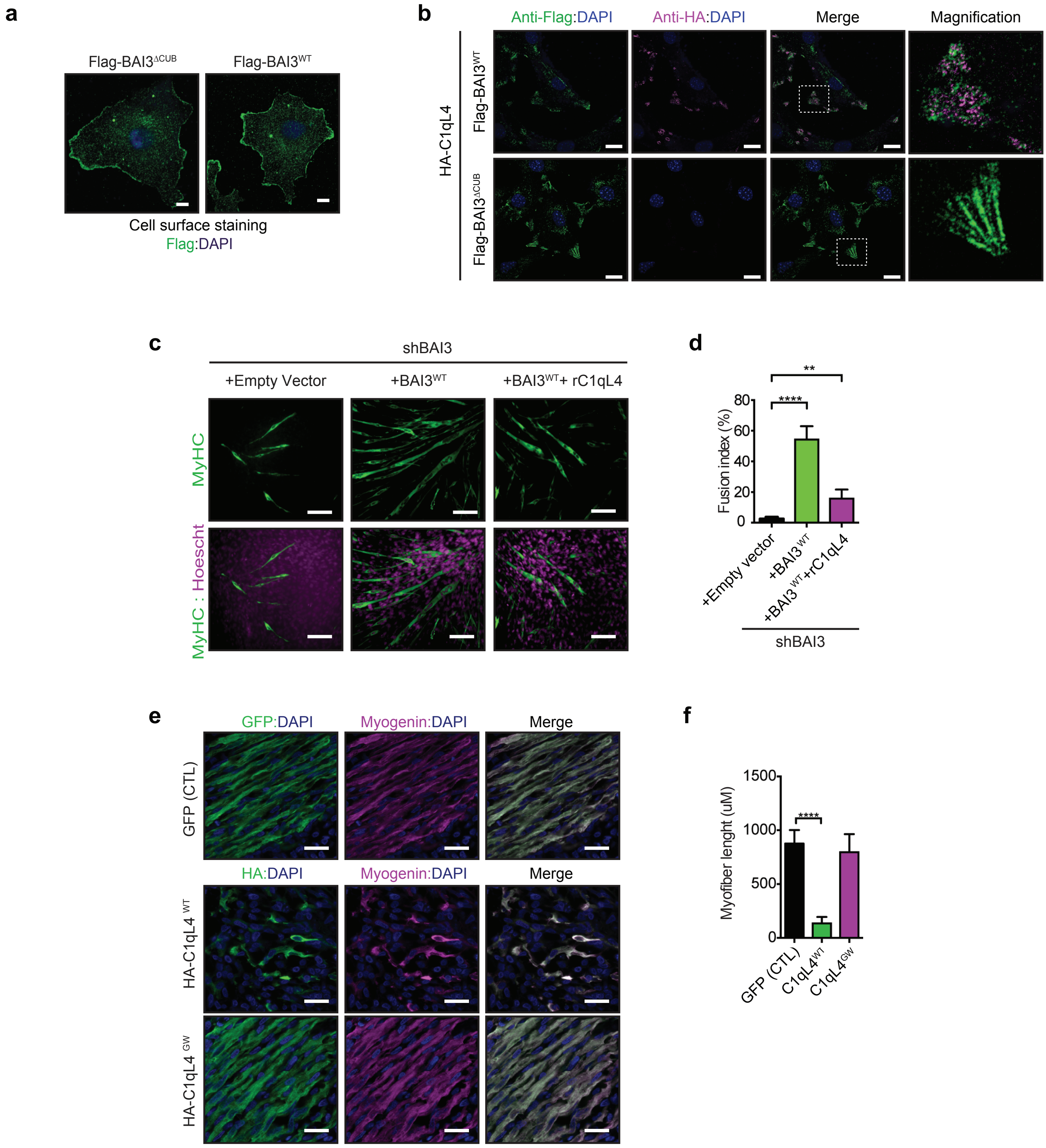

Supplementary Figure 4. C1qL4 specifically interacts with BAI3 to inhibit myoblast fusion. (a) BAI3 mutant lacking the CUB domain correctly localizes at the cell surface. C2C12 were transfected with Flag-BAI3 or Flag–BAI3$^{\Delta CUB}$. Localization of these BAI3 proteins was confirmed by staining non-permeabilized cells with an anti-Flag antibody. **(b)** C1qL4 interacts with BAI3 at the cell surface. Live cell binding assay reveals interaction of C1qL4 with Flag-BAI3 expressed at C2C12 surface. C1qL4 is not detected at the surface of cells expressing Flag-BAI3$^{\Delta CUB}$. **(c-d)** C1qL4 inhibits myoblast fusion through its interaction with BAI3. ShBAI3 cells were transfected with BAI3 to rescue myoblast fusion. Recombinant C1qL4 was added and a decrease in myoblast fusion was observed compared to BAI3 alone. **(e-f)** Expression of HA-C1qL4^{WT} or C1qL4 deficient in BAI3-binding (HA-C1ql4^{GW}) in muscle progenitors does not affect myoblast differentiation *in ovo* in chick embryos. (**f)** Length of myofibers (μm) was quantified in the indicated conditions. Myofibers were stained for Myosin Heavy Chain (MyHC, MF20 antibody (green)) and nuclei were revealed by Hoechst (purple). Error bars indicate standard deviation. Scale bar=100um. One-way ANOVA followed by a Bonferroni test was used to calculate the p values; **P<0.01, ****P<0.0001.

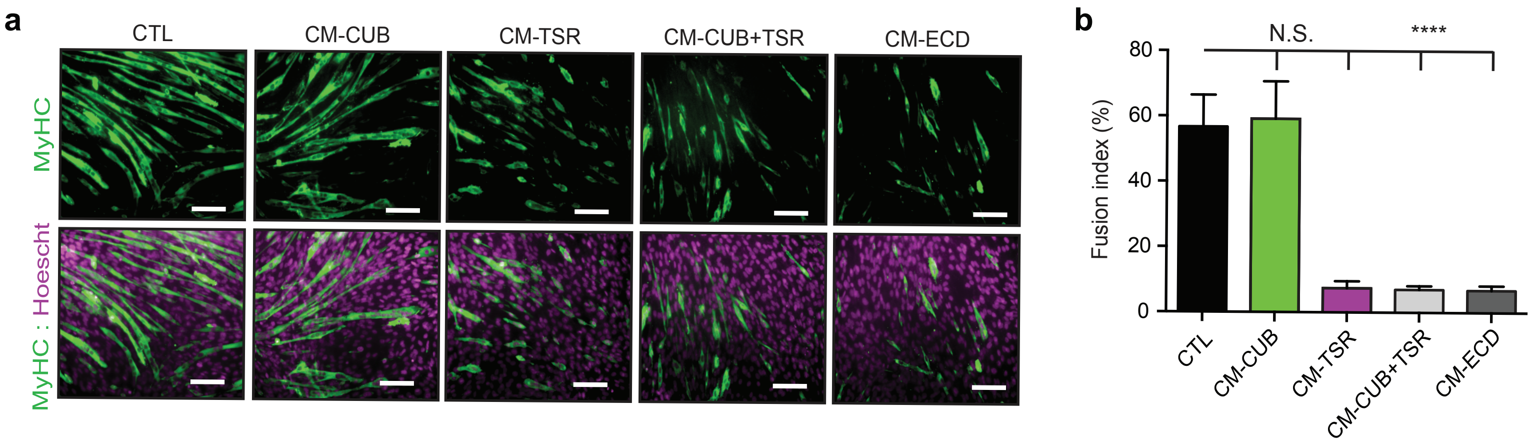

Supplementary Figure 5. The TSRs region is the minimal fragment required to inhibit myoblast fusion. (a) C2C12 cells were treated with conditioned media containing extracellular fragments of BAI3: CUB alone, TSRs alone, TSRs+CUB or the full ECD. **(b)** Quantification of experiments shown in **(a)**. Myofibers were stained for Myosin Heavy Chain (MyHC, MF20 antibody (green)) and nuclei were revealed by Hoechst (purple). Error bars indicate standard deviation. Scale bar=100um. One-way ANOVA followed by a Bonferroni test was used to calculate the p values; ****P<0.0001.

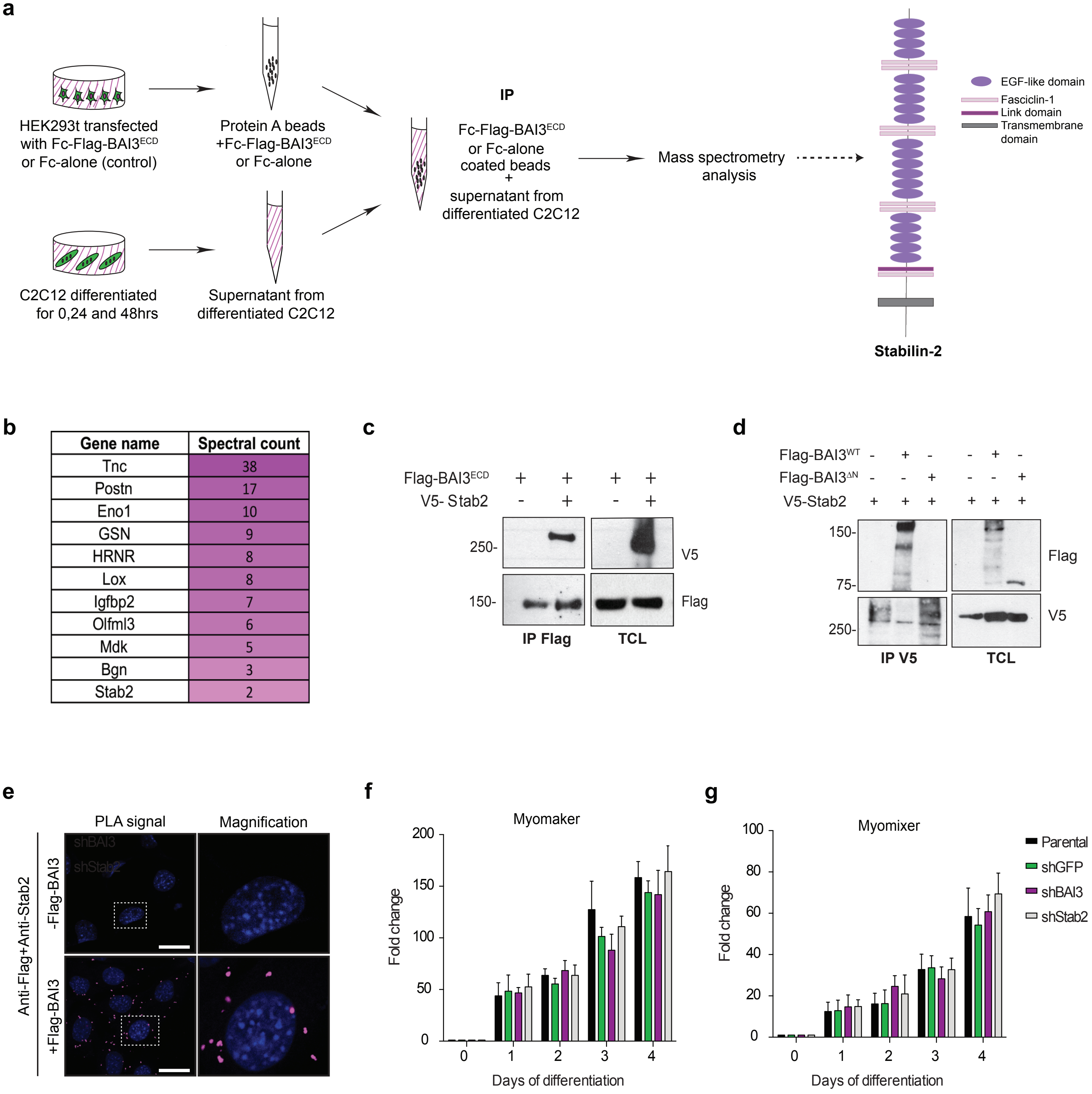

Supplementary Figure 6. The extracellular region of BAI3 interacts with Stabilin-2. (a) Affinity purification followed by mass spectrometry analyses uncover new candidate BAI3-interacting partners. **(b)** Mass spectrometry analyses reveal Stabilin-2 as a new candidate BAI3-interacting protein. **(c)** Validation of the mass spectrometry data for Stabilin-2. Flag-BAI^{ECD} is able to interact with V5-Stabilin-2. Immunoprecipitation of Flag-tagged Stabilin-2 was carried out to assess its interaction with extracellular region of BAI3. **(d)** Stabilin-2 interacts with the extracellular domain of BAI3. Immunoprecipitation of V5-Stabilin-2 demonstrates that it fails to interact with BAI3 lacking extracellular domain. **(e)** Proximity Ligation Assay (PLA) experiments reveal that tracer levels of Flag-BAI3 interacts with endogenous Stabilin-2 at cell surface of C2C12 cells (i.e. non-permeabilized cells). **(f-g)** BAI3 or Stabilin-2 are not required for the expression of Myomaker and Myomixer. Real-time Q-RT-PCR amplifications of Myomaker et Myomixer mRNAs in cells depleted of Stabilin-2 or BAI3 (4 days of differentiation). Scale bar=100um.

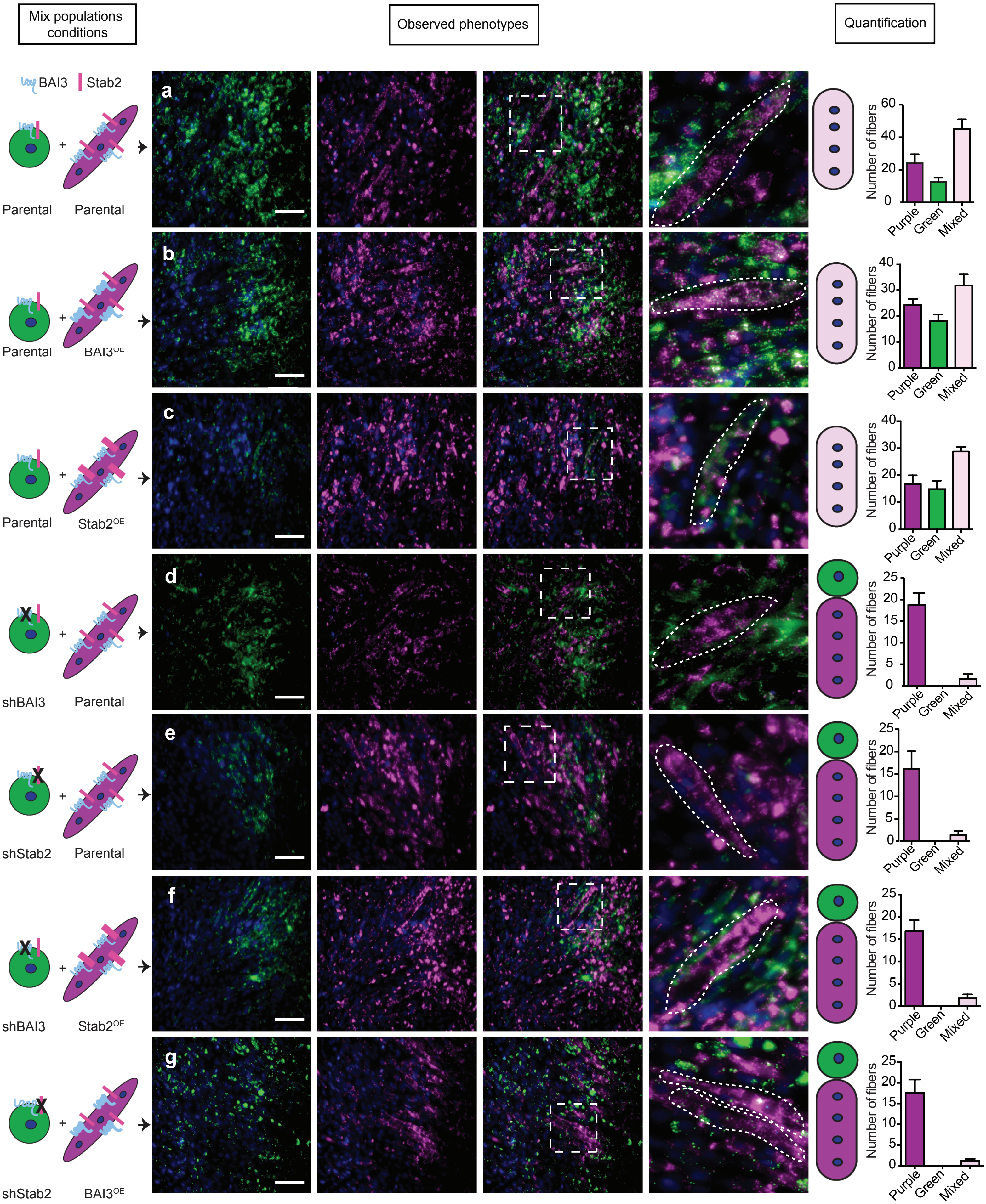

Mix populations conditions
Observed phenotypes
Quantification
BAI3
Stab2
a
b
c
d
e
f
g
Parental
Parental
Parental
BAI3OE
Parental
Stab2OE
shBAI3
Parental
shStab2
Parental
shBAI3
Stab2OE
shStab2
BAI3OE
Number of fibers
Purple
Green
Mixed

Supplementary Figure 7. BAI3 and Stabilin-2 interact in cis in secondary waves of fusion. Stabilin-2 and BAI3 are required on both myoblast and myofiber to promote cell fusion. (a) Parental C2C12 cells were stained with membrane dyes (see Methods) and differentiated for 48 hrs. Parental myoblasts were then mixed in a 1:1 ratio with formed myotubes and differentiated for another 48 hrs. **(b)** Parental C2C12 mixed with myofibers overexpressing Flag-BAI3. (**c)** Parental C2C12 (green) mixed with myofibers overexpressing V5-Stab2 (purple). Cell fusion was intact and mixed fibers were observed after differentiation. **(d)** C2C12 expressing shRNA against BAI3 (green) are mixed with parental myofibers (purple). Cells without BAI3 were unable to fuse and mix with other population after differentiation. (**e)** C2C12 expressing shRNA against Stab2 (green) were mixed with parental myofibers (purple). Mixed fibers were not observed and only cells expressing both receptors were able to fuse together after differentiation. **(f)** Cells expressing shRNA against BAI3 (green) were mixed with myofibers overexpressing V5-Stab2 (purple). (**g)** Cells expressing shRNA against Stab2 (green) were mixed with myofibers overexpressing Flag-BAI3 (purple) and no fusion was observed following differentiation. The quantification of the phenotypes was analyzed from multiple images. Scale bar=100 µm.

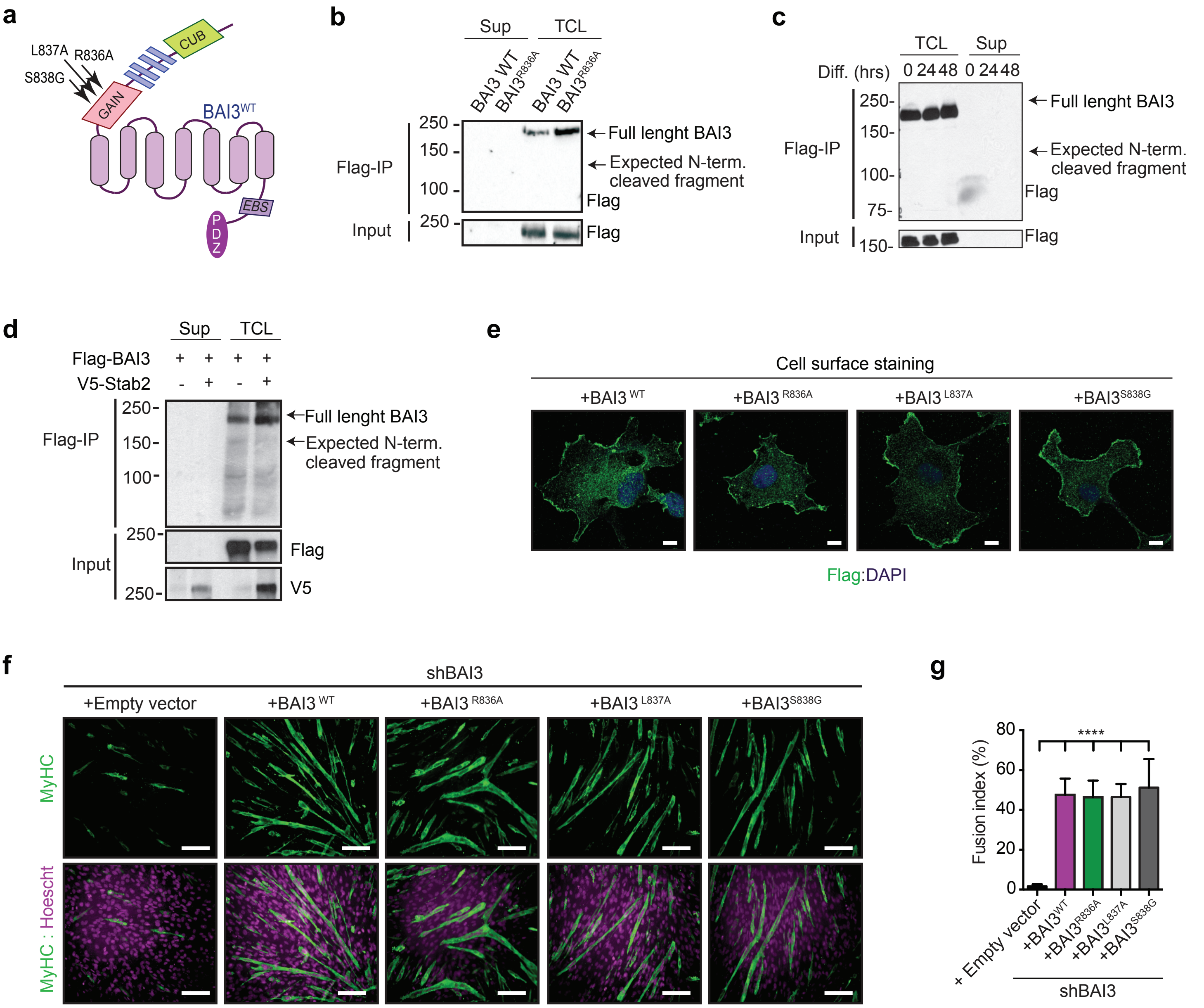

Supplementary Figure 8. BAI3 promotes myoblast fusion in an auto-proteolytic-independent manner. (a) Schematic representation of BAI3 mutants targeting 3 potential important residues for cleavage in the GAIN domain. **(b)** Overexpression of either Flag-BAI3 or a BAI3 mutant in the GAIN generates full-length proteins. Immunoprecipitation of Flag-BAI3 or Flag-BAI3^{R836A} from both the cell culture supernatants (Sup) and total cell lysates (TCL) of transfected HEK293T cells. **(c)** Exogenous Flag-BAI3 is not auto-processed during C2C12 cells differentiation. Immunoprecipitations of Flag-BAI3 from the cell culture supernatants (Sup) and total cell lysates (TCL) from differentiating C2C12 transfected with Flag-BAI3. **(d)** Stabilin-2 does not induce BAI3 auto-cleavage. Immunoprecipitations of Flag-BAI3 from cell culture supernatants (Sup) and total cell lysates (TCL) from HEK-293T transfected cells were carried out to detect BAI3 protein integrity following V5-Stabilin-2 co-expression. **(e-g)** BAI3 auto-cleavage is not required for myoblast fusion. **(e)** Mutations in the GAIN domain of BAI3 does not affect the trafficking of the receptor to the cell surface. Localization of the Flag-BAI3 proteins (WT, R836A, L837A or S838G) at the cell surface was assessed by staining the non-permeabilized cells with the anti-Flag antibody. **(f)** The myoblast fusion defect of C2C12 cells depleted of endogenous BAI3 is rescued as efficiently by Flag-BAI3 mutants in the GAIN domain in comparison to the wild-type protein. **(g)** Quantification of experiments shown in **(f)**. Myofibers were stained for Myosin Heavy Chain (MyHC, MF20 antibody (green)) and nuclei were revealed by Hoechst (purple). Error bars indicate standard deviation. Scale bar=100um. One-way ANOVA followed by a Bonferroni test was used to calculate the p values; ****$P<0.0001$.

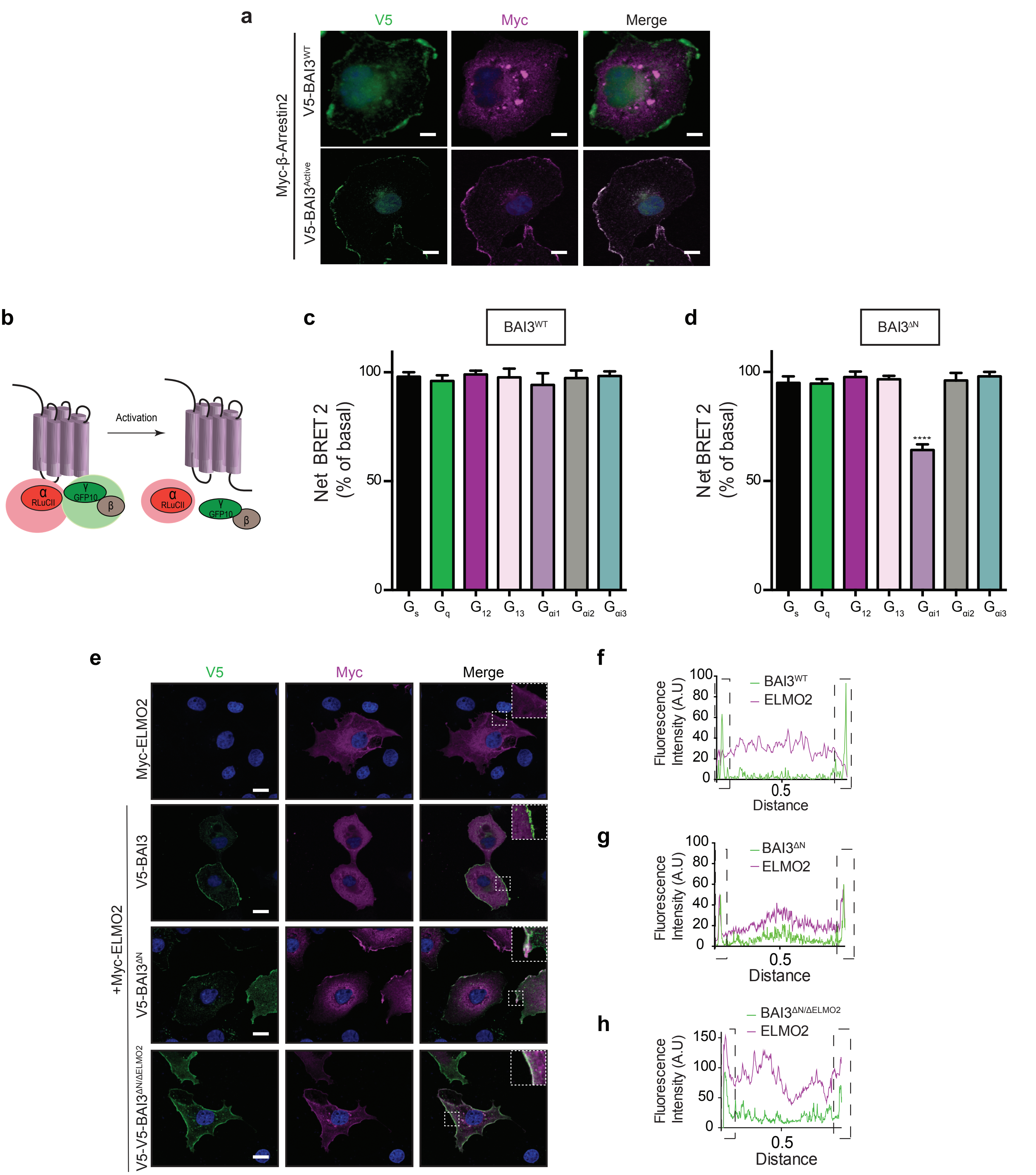

Supplementary Figure 9. BAI3 exhibits canonical GPCR activity. **(a)** Immunofluorescence experiments assessing the ability of BAI3^{WT} or BAI3$^{\Delta N}$ (green) to recruit Myc-β-Arrestin2 (purple) at the membrane of COS7 cells. **(b)** Schematic of the BRET experiment. **(c-d)** Effect of BAI3^{WT} and BAI3$^{\Delta N}$ on BRET2 signal between G-proteins Gαi1-RlucII and GFP10-Gγ2 in cells co-transfected with the untagged Gß. Net BRET2 signal is normalized to basal BRET2 signal. **(e-h)** BAI3$^{\Delta N}$ promotes the recruitment of ELMO2 at the membrane of COS7 cells. **(e)** Immunofluorescence assessing ELMO2 (purple) recruitment at the membrane in the presence of either Flag–tagged BAI3^{WT}, BAI3$^{\Delta N}$ or BAI3$^{\Delta N/\Delta ELMO2}$ (green). **(f-h)** Quantification of experiments shown in **(e)**.

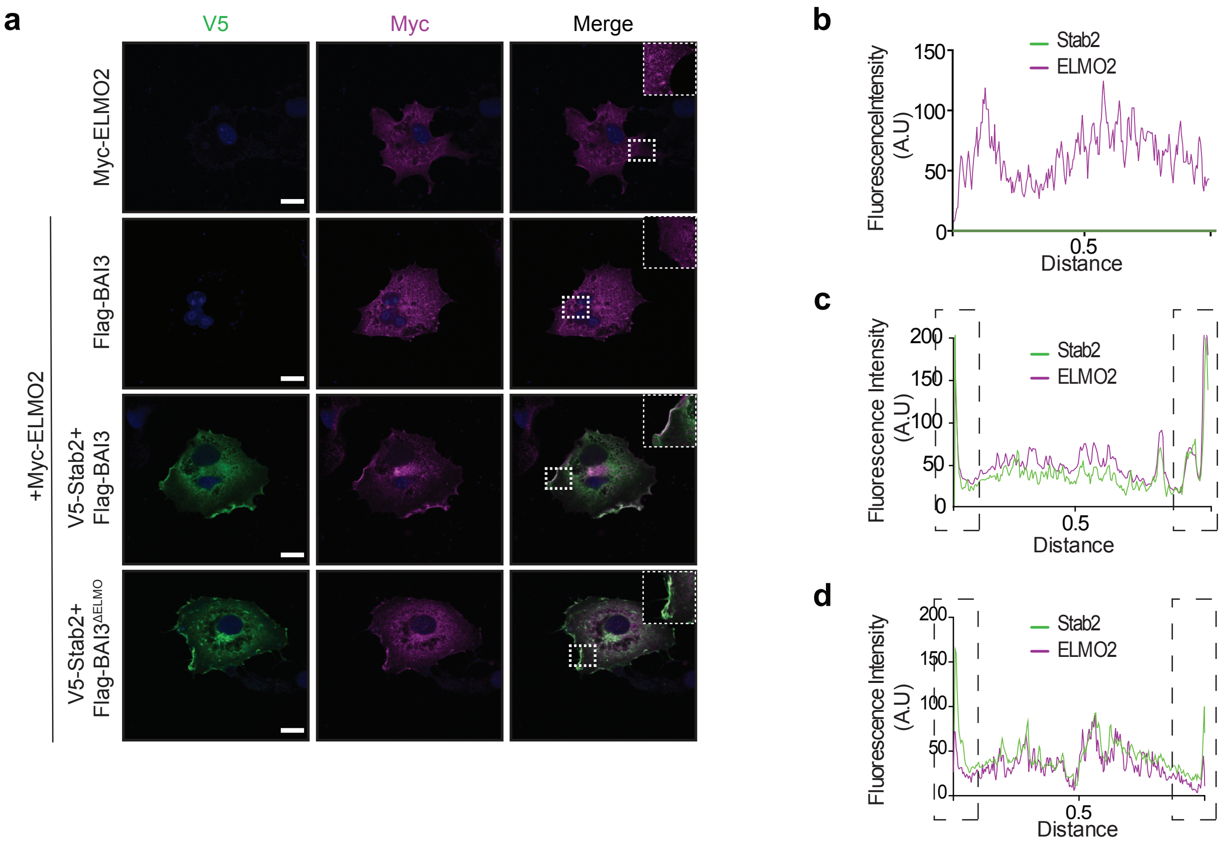

Supplementary Figure 10. Co-expression of Stabilin-2 and BAI3 promotes the recruitment of ELMO2 at the membrane. (a) Immunofluorescence experiments assessing ELMO2 (purple) recruitment at the membrane of COS7 cells in the presence of either Flag–tagged BAI3^{WT}, Stabilin-2 or BAI3$^{\Delta ELMO2}$ (green). The ELMO-binding site (EBS) of BAI3 is required for efficient membrane recruitment of Myc-ELMO2. **(b-d)** Quantification of experiments shown in **(a)**.

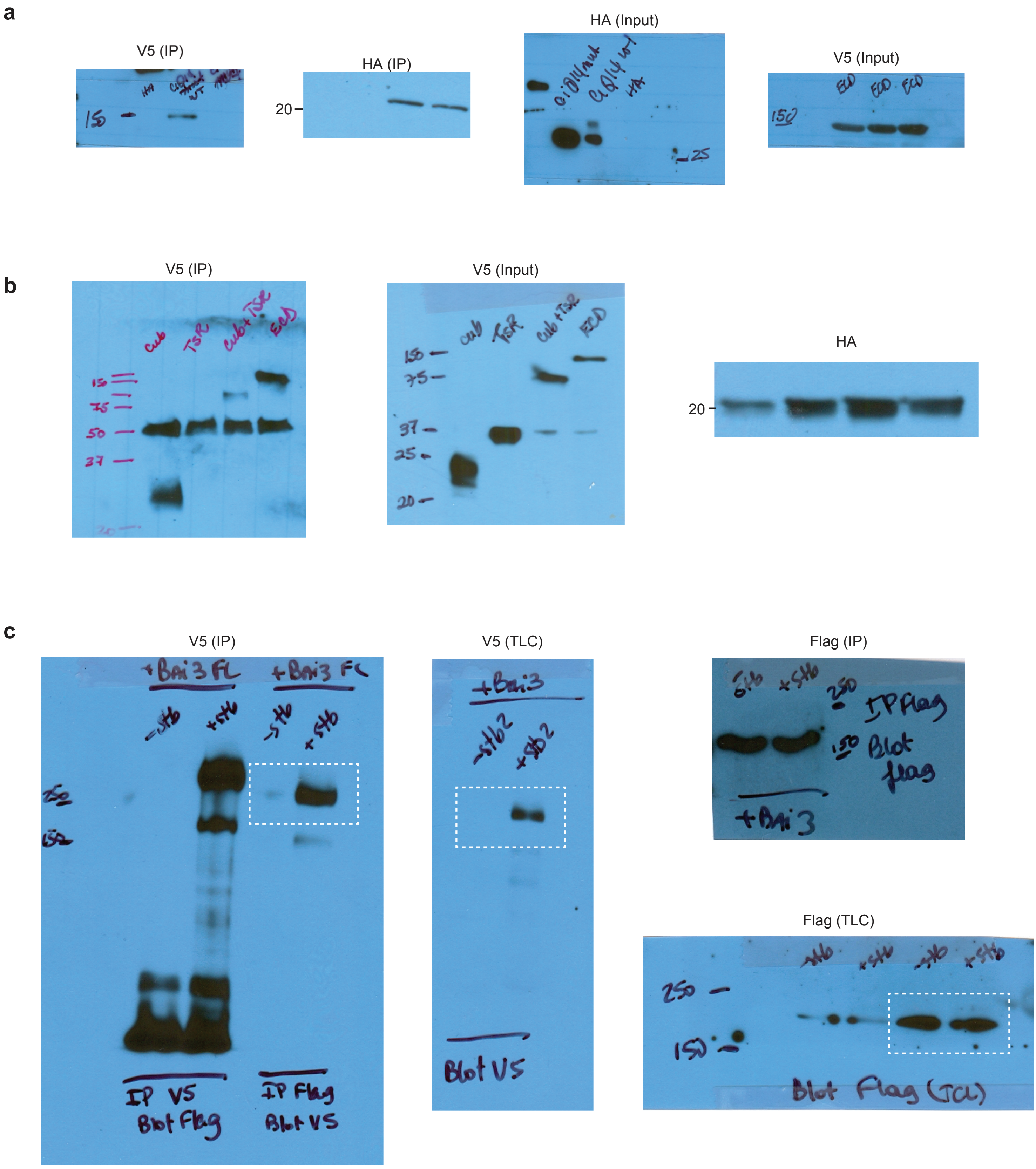

Supplementary Figure 11. Full size of the blots shown in (**a**) Figure 3a, (**b**) Figure 3f and (**c**) Figure 5g.

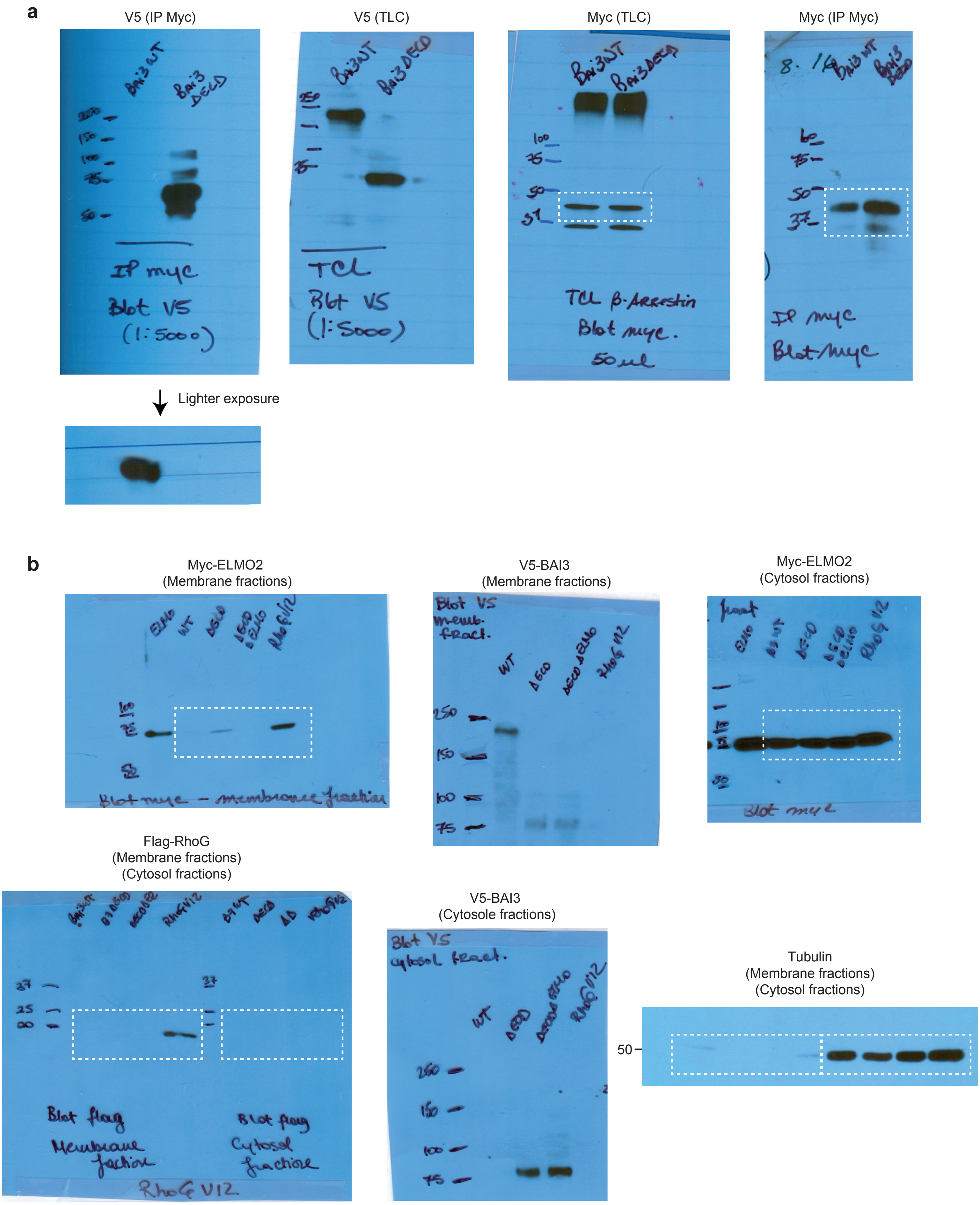

Supplementary Figure 12. Full size of the blots shown in (**a**) Figure 7a and (**b**) Figure 7i.

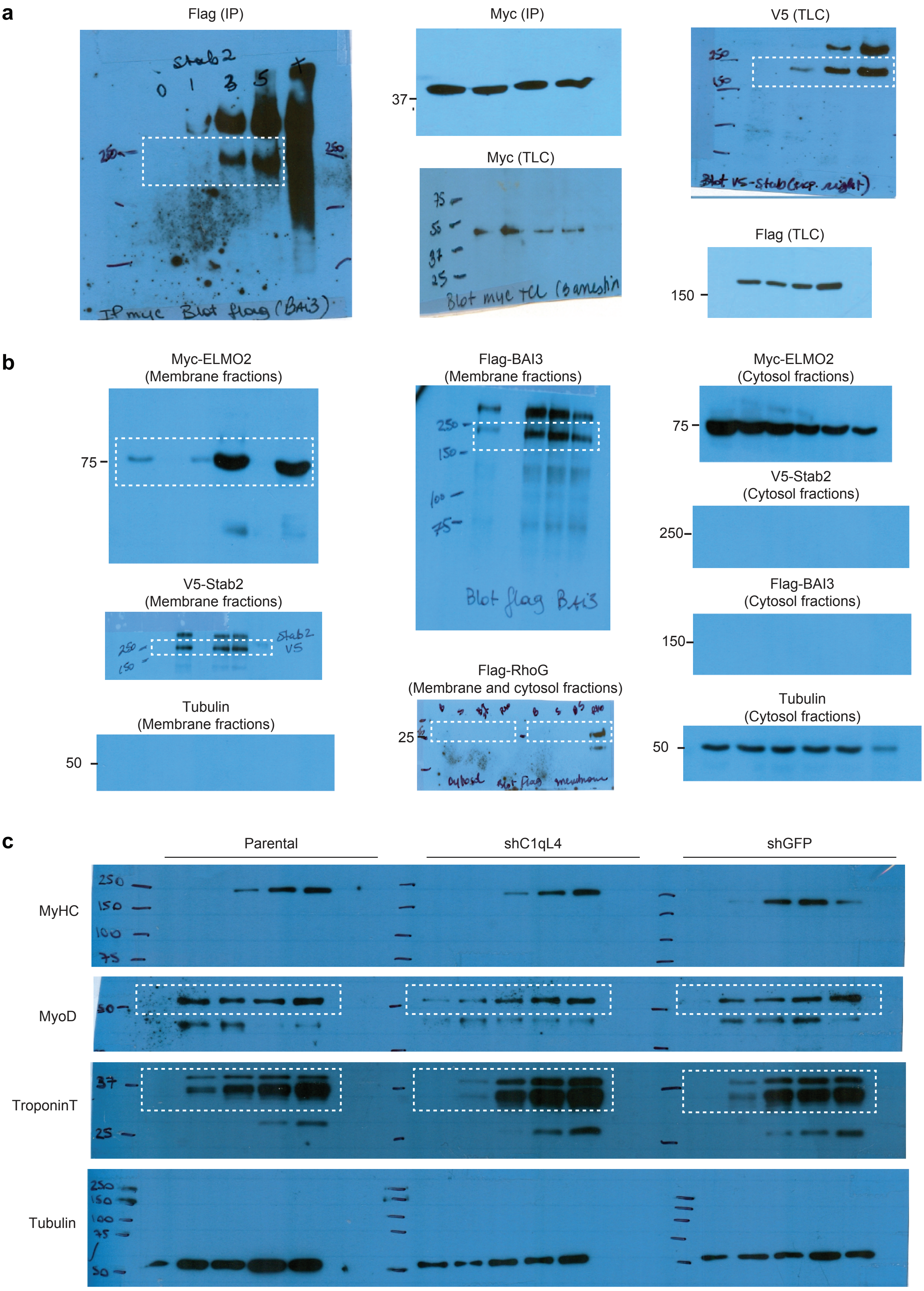

Supplementary Figure 13. Full size of the blots shown in (**a**) Figure 8a, (**b**) Figure 8f and (**c**) Supplementary Figure 2a.

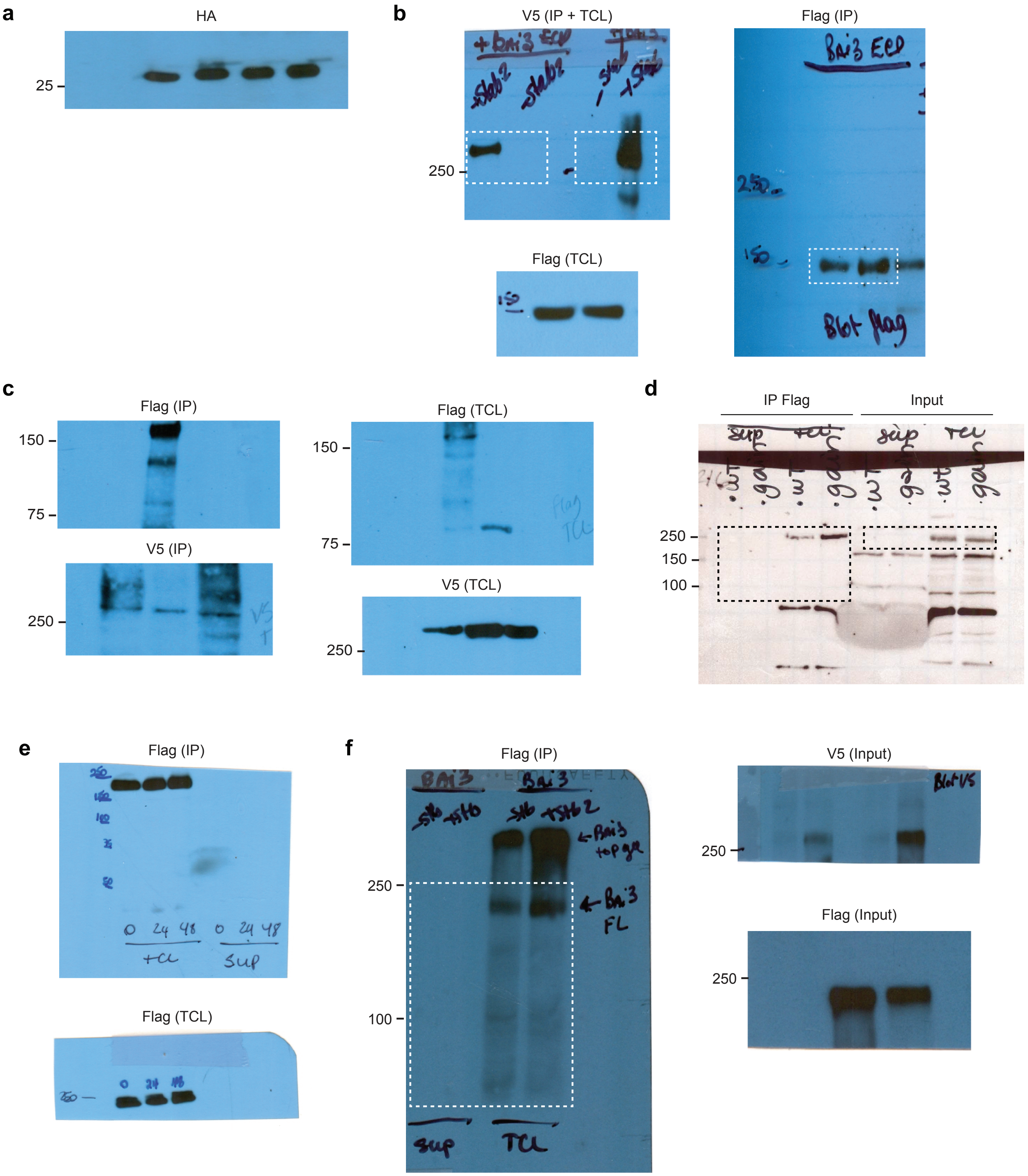

Supplementary Figure 14. Full size of the blots shown in (**a**) Supplementary Figure 3c, (**b**) Supplementary Figure 6c, (**c**) Supplementary Figure 6d, (**d**) Supplementary Figure 8b, (**e**) Supplementary Figure 8c and (**f**) Supplementary Figure 8d..

Supplementary Table 1. Various plasmids used in this study

Plasmid	Description	Purpose	Source
pCAGGS-FLAG-BAI3$^{\Delta CUB}$ -mVenus	Human BAI3 with the deletion of the CUB domain	Myoblast fusion assay	This study
pCAGGS-Flag-BAI3^{ECD}-Fc	N-terminal region of BAI3 with N-term Flag and C-term Fc-fusion	Mass spectrometry experiement	[1]
pCAGGS-Flag-BAI3^{L837A}-mVenus	Human BAI3 with a point mutation in the GAIN domain (L837A)	Myoblast fusion assay	This study
pCAGGS-Flag-BAI3^{R836A}-mVenus	Human BAI3 with a point mutation in the GAIN domain (R836A)	Myoblast fusion assay	This study
pCAGGS-Flag-BAI3^{S838G}-mVenus	Human BAI3 with a point mutation in the GAIN domain (S838G)	Myoblast fusion assay	This study
pCAGGS-Flag-BAI3$^{\Delta ELMO}$ -mVenus	Human BAI3 with a point mutation in the Elmo-binding site	Myoblast fusion assay	[2]
pCAGGS-Flag-BAI3^{WT}-mVenus	Human BAI3 with the N-term Flag and C-term mVenus	Myoblast fusion assay	[1]
pCAGGS-HA-C1qL4^{GW}	HA-C1qL4 with N-glycosylation bridge (Q191N N192S Y193T T224N K226S) in the C1q domain	Chick electroporation experiment	This study
pCAGGS-HA-C1qL4^{WT}	HA-C1qL4 WT insert in pCAGGS backbone plasmid	Chick electroporation experiment	This study
pCAGGS-V5-BAI3$^{\Delta N}$	Deletion of the N-terminus region of BAI3 to the GPS in the gain domain	BRET2 experiment, Myoblast fusion assay	This study
pCAGGS-V5-BAI3$^{\Delta N/\Delta ELMO}$	pCAGGS-V5-BAI3$^{\Delta N}$ with a point mutation in the Elmo-binding site (EBS)	Protein-protein interaction experiment	This study
pCAGGS-V5-BAI3^{WT}	BAI3 WT tagged with N-term V5 tag	BRET2 experiment	This study
pCDNA-ELMO2-RLUCII	ELMO2 tagged with RLUCII at C-terminus	BRET2 experiment	This study
pCDNA3-hC1qL1-HA	human C1qL1 with HA tag	Myoblast fusion assay	[3]
pCDNA3-mC1qL2-HA	mouse C1qL2 with HA tag	Myoblast fusion assay	[3]
pCDNA3-mC1qL3-HA	mouse C1qL3 with HA tag	Myoblast fusion assay	[3]
pCDNA3-Myc-Arrestin2	B-Arrestin2 tagged with myc	Protein-protein interaction experiment	[4]
pCDNA5-V5-Stab2	Stabilin-2 full length with V5 tag	Protein-protein interaction experiment	[5]
pDisplay-HA-C1qL4	Mouse C1qL4 with the HA tag	Protein-protein interaction experiment	[1]
pDisplay-HA-C1qL4^{GW}	C1qL4 with N-glycosylation bridge in the C1q domain	Protein-protein interaction experiment	This study
pEZY-Flag-RhoG^{G12V}	Constitutively active RhoG mutant	Protein-protein interaction experiment	
pIRES-V5-BAI1-ECD	N-terminal region of BAI1 with V5 tag	Protein-protein interaction experiment	This study
pIRES-V5-BAI2-ECD	N-terminal region of BAI2 with V5 tag	Protein-protein interaction experiment	This study
pIRES-V5-BAI3-CUB	BAI3 CUB domain	Protein-protein interaction experiment	This study
pIRES-V5-BAI3-CUB+TSR	BAI3 CUB Domain together with TSR domain	Protein-protein interaction experiment	This study
pIRES-V5-BAI3-ECD	Complete N-terminal region of BAI3	Protein-protein interaction experiment	This study
pIRES-V5-BAI3-TSR	BAI3 TSR domain	Protein-protein interaction experiment	This study

Supplementary Table 2. Primers used in this study

	Plasmid/Purpose	Forward	Reverse
C1qL 1	RT-PCR	5' ACG AGG TGC TCA AGT TTG 3'	5' ATT TGT TGC TGT TGC CCC 3'
C1qL 2	RT-PCR	5' TAC GAG GTG CTC AAG TTC G 3'	5' TGT TAT TGT TGC CGC CGT G 3'
C1qL 3	RT-PCR	5' GGT TTC CGA GAT TGG TTA CC 3'	5' CGC ACC TGG TTG TTT TTG 3'
C1qL 4	RT-PCR	5' AGG GTT ATG AAG TGT TGC G 3'	5' GGG TAG ATG ATG AAT CCT GAG AAG 3'
β-Actin	RT-PCR	5' CCT AGG CAC CAG GGT GTG AT 3'	5' GCC TCG TCA CCC ACA TAG GA 3'
Cloning of the N-terminus of BAI1	pIRES-V5-BAI1-ECD	5' C GGT CTC GAT TCT ACG GCC GCC GGA GCA GAC GC 3'	5' CT GCA GAA TTC TCA GGG CCT GCA GGT GCG CGT 3'
Cloning of the N-terminus of BAI2	pIRES-V5-BAI2-ECD	5' C GGT CTC GAT TCT ACG CTG GCC ACG GCC TTC GAC 3'	5' GTC GAA GGC CGT GGC CAG CGT AGA ATC GAG ACC G 3'
Cloning of mC1qL4 in pCAGGS vector	pCAGGS-HA-C1qL4^{WT}	5' GAA TTC TAC CCA TAC GAT GTT CCA GAT TAC GCT 3'	5' GGT ACC TCA GTC CGG GTA GAT GAT GA 3'
Cloning of CUB domain	pIRES-V5-BAI3-CUB	5' CCT CGG TCT CGA TTC TAC GCG TAG TAC TTG TTC AAC TTT GGT GAA GGG AGT CAT 3'	5' GGT GAT GGT GAT GGT GAC GCG TCT CGA GGT TCA ATA CCA AAA ACT CAA AAA AAG ATT TCT GAT CTT CT 3'
Cloning of TSR domain	pIRES-V5-BAI3-TSR	5' CCT CGG TCT CGA TTC TAC GCG TAG TAC TGT GGA AGA GTG GTC CCA GT 3'	5' GGT GAT GGT GAT GGT GAC GCG TCT CGA GAG GGC ATC GCT GCT CAT 3'
Generation of mC1qL 4 Q191N N192S Y193T mutant	pDisplay-HA-C1qL4^{GW}	5' CGC CAT TGC TCA GGA TGC GGA TAA TAG CAC CGA CTA TGC CAG CAA CAG CG 3'	5' CGC TGT TGC TGG CAT AGT CGG TGC TAT TAT CCG CAT CCT GAG CAA TGG CG 3'
Generation of mC1qL 4 T224N K226S mutant	pDisplay-HA-C1qL4^{GW}	5' GGT GCA CGG CGG GAA CAA CAA TAG CTA CAG CAC CTT CTC AGG 3'	5' CCT GAG AAG GTG CTG TAG CTA TTG TTG TTC CCG CCG TGC ACC 3'
Cloning of hBai3 R836A mutant	pCAGGS-Flag-BAI3^{R836A}-mVenus	5' CAT CCC ATA CGA AAT GCT TAT GTG ATA GTC TCT CTA CCT TC 3'	5' GAA GGT AGA GAG ACT ATC ACA TAA GCA TTT CGT ATG GGA TG 3'
Cloning of hBai3 S838G mutant	pCAGGS-Flag-BAI3^{S838G}-mVenus	5' ATG CTT ATG TGA TCG TCT CGG TAC CTT CGC CAT TTT GGC T 3'	5' AGC CAA AAT GGC GAA GGT ACC GAG ACG ATC ACA TAA GCA T 3'
Cloning of hBai3 L837A mutant	pCAGGS-Flag-BAI3^{L837A}-mVenus		
Cloning of hBai3$^{\Delta N}$	pIRES-V5-BAI3$^{\Delta N}$	5' CCT CGG TCT CGA TTC TAC GCG TAG TAC TAT AAT CAT GGA ATC CTC TGG CAC 3'	5' GGT GAT GGT GAT GGT GAC GCG TCT CGA GAA CTT CTG TTT GAA AGT CAC C 3'
Cloning of mELMO2 tagged with RLUCII (for BRET2)	pCDNA-ELMO2-RLUCII	5' GGA GAC CCA AGC TGG CTA GCA TGC CGC CTC CGT CTG 3'	5' GTC ATG GTG GCG GGA AGC TTG CCA TAG TGA TAG ACG AAG TCA TAG CT 3'

Supplementary Table 2. Primers used in this study (continued)

	Plasmid/Purpose	Forward	Reverse
mC1qL 1	Q-RT-PCR	5’ ATG AGG GTT ACG AGG TGC TC 3’	5’ CAT GAG GAC GTG GTA GGT GA 3’
mC1qL 2	Q-RT-PCR	5’ GGA CTG CAG TTG ACA ACC AG 3’	5’ GCT CTT GAG TCC CAC GTA GA 3’
mC1qL 3	Q-RT-PCR	5’ ATG TGG GCT GAT CTC TGC AA 3’	5’ AGG TGA AGG ACC ACA CTG TT 3’
mC1qL 4	Q-RT-PCR	5’ ATG TGG GCC GAT CTG ATG AA 3'	5' CTC TAG GTG CAG AAT GAC GCT 3'
TATA Box	Q-RT-PCR	5’ GCT GAA TAT AAT CCC AAG CGA TTT 3’	5’ GCA GTT GTC CGT GGC TCT CT 3’
MyoD	Q-RT-PCR	5’ GGC GAC TCA GAT GCA TCC A 3’	5’ CTG TAA TCC ATC ATG CCA TCA GA 3’
Myogenin	Q-RT-PCR	5’ TCC CAA CCC AGG AGA TCA TTT 3’	5’ GCA GAT TGT GGG CGT CTG TA 3’
MyHC4	Q-RT-PCR	5’ GCA GGA CTT GGT GGA CAA AC 3’	5’ ACT TGG CCA GGT TGA CAT TG 3’
Myomaker	Q-RT-PCR	5’ ATC GCT ACC AAG AGG CGT T 3’	5’ CAC AGC ACA GAC AAA CCA GG 3’
Myomixer	Q-RT-PCR	5’ ATG TCT TGG GAG CTC AGT CG 3’	5’ ACC AGC TTT CAT GCC AGA AG 3’

Supplementary Table 3. Short-hairpin RNAs (shRNAs) used in C2C12 and Sol8 knockdown experiments

	Forward	Reverse
shRNA against mC1qL4 (Pos 655)	5' GAT CCG CAC GGC GGG AAC ACC AAT TTC AAG AGA ATT GGT GTT CCC GCC GTG CTT TTT AGA TCT G 3'	5' AAT TCA GAT CTA AAA AGC ACG GCG GGA ACA CCA ATT CTC TTG AAA TTG GTG TTC CCG CCG TGC G 3'
shRNA against mC1qL4 (Pos 560)	5' GAT CCG GAT CAG AAC TAC GAC TAT TTC AAG AGA ATA GTC GTA GTT CTG ATC CTT TTT AGA TCT G 3'	5' AAT TCA GAT CTA AAA AGG ATC AGA ACT ACG ACT ATT CTC TTG AAA TAG TCG TAG TTC TGA TCC G 3'
shRNA against mC1qL4 (Pos 582)	5' GAT CCA CTA TGC CAG CAA CAG CGT CAT TTT CAA GAG AAA TGA CGC TGT TGC TGG CAT AGT TTT TTA GAT CTG 3'	5' AAT TCA GAT CTA AAA AAC TAT GCC AGC AAC AGC GTC ATT TCT CTT GAA AAT GAC GCT GTT GCT GGC ATA GTG 3'
shRNA against mStab2 (pos 6248)	5' CCC ATG TCA TAA GAA TGC GAA TTT CAA GAG AAA TGA CGC TGT TGC TGG CAT AGT TTT TTA GAT CTG 3'	5'CAGATCTAAAAAACTATGCCAGCAACAGCGTCATTTC TCTTGAAATTCGCATTCTTATGACATGGG 3'

Supplementary References

1. Bolliger, M. F., Martinelli, D. C. & Sudhof, T. C. The cell-adhesion G protein-coupled receptor BAI3 is a high-affinity receptor for C1q-like proteins. *Proc Natl Acad Sci U S A* **108**, 2534-2539, (2011).
2. Hamoud, N., Tran, V., Croteau, L. P., Kania, A. & Cote, J. F. G-protein coupled receptor BAI3 promotes myoblast fusion in vertebrates. *Proc Natl Acad Sci U S A* **111**, 3745-3750, (2014).
3. Seldin, M. M., Peterson, J. M., Byerly, M. S., Wei, Z. & Wong, G. W. Myonectin (CTRP15), a novel myokine that links skeletal muscle to systemic lipid homeostasis. *J Biol Chem* **287**, 11968-11980, (2012).
4. Quoyer, J. *et al.* Pepducin targeting the C-X-C chemokine receptor type 4 acts as a biased agonist favoring activation of the inhibitory G protein. *Proc Natl Acad Sci U S A* **110**, E5088-5097, (2013).
5. Hare, A. K. & Harris, E. N. Tissue-specific splice variants of HARE/Stabilin-2 are expressed in bone marrow, lymph node, and spleen. *Biochem Biophys Res Commun* **456**, 257-261, (2015).
